# Epidemiology of pediatric schistosomiasis in hard-to-reach areas and populations: A scoping review protocol

**DOI:** 10.12688/f1000research.126884.2

**Published:** 2023-01-20

**Authors:** Phyllis Munyiva Isaiah, Marta Sólveig Palmeirim, Peter Steinmann

**Affiliations:** 1 Swiss Centre for International Health, Swiss Tropical and Public Health Institute, Allschwil, Switzerland; 2 University of Basel, Basel, Switzerland

**Keywords:** Schistosomiasis, Prevalence, Epidemiology, Pre-School Aged Children, Pediatric, Hard-to-reach, Praziquantel

## Abstract

**Background:** Schistosomiasis is a neglected tropical disease (NTD) that affects millions of people. Children are the most vulnerable group to developing overt disease. An estimated 779 million people are at risk of schistosomiasis and 50 million preschool-age children (PSAC) need treatment. PSAC are not currently targeted by national chemotherapy campaigns due to a lack of suitable pediatric formulations of praziquantel. The Pediatric Praziquantel Consortium has developed an orally dispersible praziquantel formulation (arpraziquantel) and is facilitating its adoption for schistosomiasis control by endemic countries through the ADOPT program – an implementation research program that paves the way for the large-scale delivery of the child-friendly formulation to treat schistosomiasis in preschool-aged children in endemic countries. A key challenge for comprehensive NTD control including schistosomiasis is reaching all at-risk populations, including those hard to reach. Main access barriers include geographic, social and economic conditions.

**Objective** **:** This scoping literature review aims to document the epidemiology of schistosomiasis in children under 6 years of age living in hard-to-reach areas and populations.

**Methods** **:** This review will adopt the five-stage scoping review process of identifying the research question, identifying relevant studies, study selection, charting data and collating, summarizing and reporting results. Electronic databases including Medline, Web of Science, Embase (Ovid), LILACS and African Journals OnLine (AJOL) will be searched for relevant articles. Two independent reviewers will screen identified articles using a two-stage approach of reviewing the title/abstract and then the full text of provisionally retained articles. Relevant literatures will be downloaded into EndNote X9 to maintain and manage citation and facilitate the overall review process. A meta-analysis will be conducted if indicated.

**Relevance** **: **The results will provide insights into the burden of schistosomiasis among marginalized PSAC, aiming to produce evidence on the need for inclusion of this population when designing the expansion of preventive chemotherapy programs.

## Abbreviations and acronyms

DALYs: Disability-Adjusted Life Years

NTD: Neglected Tropical Disease

PSAC: Preschool-aged Children

*S. haematobium*: *Schistosoma haematobium*

*S. mansoni*: *Schistosoma mansoni*

SAC: School-aged children

SDG: Sustainable Development Goal

## Introduction

Schistosomiasis is a neglected tropical disease (NTD) that affects millions of people worldwide. It is estimated that 779 million people are at risk of developing this disease. ^1^^,^^2^ Children are the most vulnerable group to developing overt disease with 50 million preschool-age (PSAC) children in need of treatment. ^3^ Adults are most affected by the consequences of chronic infection. ^4^ Schistosomiasis is cause by an infection with a *Schistosoma* species parasite. ^5^ There are several species, the most common being *Schistosoma mansoni* and *Schistosoma haematobium.* In a human host, the adult parasites release eggs into the environment through faeces ( *S. mansoni*) and urine ( *S. haematobium*). An intermediate host snail living in stagnant water is involved in the transmission cycle. Human infections take place through skin penetration when in contact with stagnant water where *Schistosoma* spp. larvae are present. The disease mainly affects the poorest communities without access to safe water and improved sanitation, or exposed to water during occupational and domestic activities. ^6^^,^^7^ Symptoms of chronic schistosomiasis include anaemia, cognitive impairment, deficits in linear growth leading to chronic under-nutrition (stunting) as well as acute under-nutrition (wasting), ^5^ genital lesions, ^8^ and irreversible organ damage as a result of fibrosis. ^9^ The morbidity caused by schistosomes is commonly associated with moderate-to-heavy intensities of infection as measured by the density of excreted eggs, and is progressive. ^10^ In addition to these health consequences, schistosomiasis is associated with negative economic and social impacts. ^11^ Estimates indicate that schistosomiasis causes an annual loss of 4.5 million disability-adjusted life years (DALYs). ^12^

Although NTD control programs have been established in many endemic countries, the rolling out of schistosomiasis control and elimination components is still limited. ^13^ Periodic deworming with praziquantel, the strategy recommended by WHO for the control of schistosomiasis, is available primarily to school-aged children (SAC; 5-15 years old) who can be reached efficiently through school-based programs. ^10^ In addition to previous studies, ^14^ the 2022 WHO guideline on control and elimination of human schistosomiasis ^15^ has identified important treatment gaps in this strategy, including that infected PSAC are largely left untreated.

This could be because for a long time the PSAC group has been categorized as a low risk group for schistosomiasis infection ^16^ and its impact on the health of this age group, although unknown, is often considered negligible. ^17^ In addition, logistical and operational difficulties in collecting samples from PSAC for diagnosis, especially in hard-to-reach areas, lack of sensitive diagnostics for light schistosomiasis infections and a paucity of data on risk factors in PSAC have further biased schistosomiasis research to focus on SAC and adults over the years. ^16^ Lastly, current donations of the main drug to treat schistosomiasis – praziquantel – are restricted to SAC.

Treatment equity is not currently achieved for schistosomiasis control as hundreds of millions of the worlds’ most vulnerable, most disadvantaged people, including PSAC, are still left behind. This is true especially for people whose incomes are below the federal poverty threshold, who live in vulnerable social and economic situation such as undocumented persons, socially excluded groups due to language, religious and other societal barriers ^18^ and in the remotest, hardest to reach parts of endemic countries. These hard-to-reach populations are often ethnic minorities, island and fishing communities and migrant populations and other minority or marginalized populations, hindering the attainment of the Sustainable Development Goal (SDG) 3 and the commitment of global leaders to ensure that “no one is left behind” from development progress. ^7^^,^^19^

### Objective

This scoping literature review aims to document the epidemiology of pediatric schistosomiasis (children under 7 years of age) living in hard-to-reach areas and populations.

### Review question

The condition, context, and population (CoCoPop) framework to inform the review objective is shown in Table 1.

**Table 1.** Condition, context, and population framework.

Condition	Context	Population
Schistosomiasis infection	Hard-to-reach populations	Children under 7 years

### Definition of hard-to-reach areas and populations

In this review, hard-to-reach areas and populations are defined following Shaghaghi *et al*., as: (i) hard-to-reach areas, those living in remote physical and geographical location (e.g. migrants/island and fishing communities/nomads); (ii) hard-to-reach populations; those living in vulnerable social and economic situations such as minority groups, undocumented persons, socially excluded groups due to language and religious barriers. ^18^

### Expected outcome

The aim is to produce evidence on the need for inclusion of this population when designing the expansion of preventive chemotherapy to also cover those living in hard-to-reach areas. This is in accordance with the WHO guideline of 2022 on control and elimination of human schistosomiasis ^15^ recommendation on expansion of preventive chemotherapy to cover all in need.

## Methods

This review will adopt the five-stage scoping review process guideline recommended by Arksey *et. al.,* ^20^ taking into consideration the modifications recommended by Peters *et al.* ^21^

*Evidence searches*

Electronic literature databases including Medline, Web of Science, Embase (Ovid), LILACS and African Journals OnLine (AJOL) will be searched for published scientific studies on pediatric schistosomiasis in hard-to-reach areas, using a pre-determined search strategy ( Table 2). With the guidance of a librarian (University Medical Library - University of Basel), we first developed and optimized a search strategy for PubMed. This search was then translated using the SR-accelerator tool ^22^ developed by BOND university to generate the equivalent search terms for Embase (Ovid) and Web of Science.

**Table 2.** Searching databases and strategies.

**a.) PubMed Search Query** (Schistosomiasis [Mesh] OR Schistosom*[tiab] OR Bilharzi*[tiab] OR “blood fluke*”[tiab] OR “snail fever*”[tiab] OR “Katayama fever*”[tiab]) AND (Child [Mesh] OR child [tw] OR children [tw] OR Infant [Mesh] OR infan*[tw] OR newborn*[tw] OR new-born*[tw] OR baby [tw] OR babies [tw] OR suckling*[tw] OR toddler*[tw] OR childhood [tw] OR schoolchild*[tw] OR childcare [tw] OR child-care [tw] OR young [ti] OR youngster*[tw] OR preschool [tw] OR pre-school [tw] OR kid [tw] OR kids [tw] OR boy [tw] OR boys [tw] OR girl*[tw] OR pre-adolescen*[tw] OR schoolage*[tw] OR school-age*[tw] OR schoolboy*[tw] OR schoolgirl*[tw] OR Pediatrics [Mesh] OR Pediatric*[tw] OR Paediatric*[tw] OR (child [all] NOT child [au]) OR children*[all] OR schoolchild*[all] OR “under 5”[tw] OR “<5 year*”[all] OR “<=5 year*”[all] OR infan*[all] OR pediat*[all] OR paediat*[all] OR neonat*[all] OR toddler*[all] OR preteen*[all] OR newborn*[all] OR postneonat*[all] OR postnatal*[all] OR puberty [all] OR preschool*[all] OR suckling*[all] OR juvenile [all] OR “new born*”[all] OR new-born*[all] OR neo-nat*[all] OR neonat*[all] OR perinat*[all] OR underag*[all] OR “under age”[all] OR “under aged”[all] OR youth*[all] OR kinder*[all] OR pubescen*[all] OR prepubescen*[all] OR prepuberty [all] OR “school age”[all] OR “stratified by age”[all] OR schoolage [all] OR “school ages”[all] OR schoolage*[all] OR “one year old”[ti] OR “two year old”[ti] OR “three year old”[ti] OR “four year old”[ti] OR “five year old”[ti] OR “six year old”[ti] OR “1 year old”[ti] OR “2 year old”[ti] OR “3 year old”[ti] OR “4 year old”[ti] OR “5 year old”[ti] OR “6 year old”[ti] OR “two years old”[ti] OR “three years old”[ti] OR “four years old”[ti] OR “five years old”[ti] OR “six years old”[ti] OR “2 years old”[ti] OR “3 years old”[ti] OR “4 years old”[ti] OR “5 years old”[ti] OR “6 years old”[ti]) NOT (animals [Mesh] NOT humans [Mesh])
**b.) Web of Science Search Query** (ALL=Schistosomiasis OR (TI=Schistosom* OR AB=Schistosom*) OR (TI=Bilharzi* OR AB=Bilharzi*) OR (TI="blood fluke*" OR AB="blood fluke*") OR (TI="snail fever*" OR AB="snail fever*") OR (TI="Katayama fever*" OR AB="Katayama fever*")) AND (ALL=Child OR ALL=child OR ALL=children OR ALL=Infant OR ALL=infan* OR ALL=newborn* OR ALL=new-born* OR ALL=baby OR ALL=babies OR ALL=suckling* OR ALL=toddler* OR ALL=childhood OR ALL=schoolchild* OR ALL=childcare OR ALL=child-care OR TI=young OR ALL=youngster* OR ALL=preschool OR ALL=pre-school OR ALL=kid OR ALL=kids OR ALL=boy OR ALL=boys OR ALL=girl* OR ALL=pre-adolescen* OR ALL=schoolage* OR ALL=school-age* OR ALL=schoolboy* OR ALL=schoolgirl* OR ALL=Pediatrics OR ALL=Pediatric* OR ALL=Paediatric* OR (ALL=child NOT AU=child) OR ALL=children* OR ALL=schoolchild* OR ALL="under 5" OR ALL="<5 year*" OR ALL="<=5 year*" OR ALL=infan* OR ALL=pediat* OR ALL=paediat* OR ALL=neonat* OR ALL=toddler* OR ALL=preteen* OR ALL=newborn* OR ALL=postneonat* OR ALL=postnatal* OR ALL=puberty OR ALL=preschool* OR ALL=suckling* OR ALL=juvenile OR ALL="new born*" OR ALL=new-born* OR ALL=neo-nat* OR ALL=neonat* OR ALL=perinat* OR ALL=underag* OR ALL="under age" OR ALL="under aged" OR ALL=youth* OR ALL=kinder* OR ALL=pubescen* OR ALL=prepubescen* OR ALL=prepuberty OR ALL="school age" OR ALL="stratified by age" OR ALL=schoolage OR ALL="school ages" OR ALL=schoolage* OR TI="one year old" OR TI="two year old" OR TI="three year old" OR TI="four year old" OR TI="five year old" OR TI="six year old" OR TI="1 year old" OR TI="2 year old" OR TI="3 year old" OR TI="4 year old" OR TI="5 year old" OR TI="6 year old" OR TI="two years old" OR TI="three years old" OR TI="four years old" OR TI="five years old" OR TI="six years old" OR TI="2 years old" OR TI="3 years old" OR TI="4 years old" OR TI="5 years old" OR TI="6 years old") NOT (ALL=animals NOT ALL=humans)
**C.) Embase (Ovid) Search Strategy** (exp Schistosomiasis/ OR Schistosom*.tw. OR Bilharzi*.tw. OR “blood fluke*”.tw. OR “snail fever*”.tw. OR “Katayama fever*”.tw.) AND (exp Child/ OR child.mp. OR children.mp. OR exp Infant/ OR infan*.mp. OR newborn*.mp. OR new-born*.mp. OR baby.mp. OR babies.mp. OR suckling*.mp. OR toddler*.mp. OR childhood.mp. OR schoolchild*.mp. OR childcare.mp. OR child-care.mp. OR young.ti. OR youngster*.mp. OR preschool.mp. OR pre-school.mp. OR kid.mp. OR kids.mp. OR boy.mp. OR boys.mp. OR girl*.mp. OR pre-adolescen*.mp. OR schoolage*.mp. OR school-age*.mp. OR schoolboy*.mp. OR schoolgirl*.mp. OR exp Pediatrics/OR Pediatric*.mp. OR Paediatric*.mp. OR (child.af. NOT child.au.) OR children*.af. OR schoolchild*.af. OR “under 5”.mp. OR “<5 year*”.af. OR “<=5 year*”.af. OR infan*.af. OR pediat*.af. OR paediat*.af. OR neonat*.af. OR toddler*.af. OR preteen*.af. OR newborn*.af. OR postneonat*.af. OR postnatal*.af. OR puberty.af. OR preschool*.af. OR suckling*.af. OR juvenile.af. OR “new born*”.af. OR new-born*.af. OR neo-nat*.af. OR neonat*.af. OR perinat*.af. OR underag*.af. OR “under age”.af. OR “under aged”.af. OR youth*.af. OR kinder*.af. OR pubescen*.af. OR prepubescen*.af. OR prepuberty.af. OR “school age”.af. OR “stratified by age”.af. OR schoolage.af. OR “school ages”.af. OR schoolage*.af. OR “one year old”.ti. OR “two year old”.ti. OR “three year old”.ti. OR “four year old”.ti. OR “five year old”.ti. OR “six year old”.ti. OR “1 year old”.ti. OR “2 year old”.ti. OR “3 year old”.ti. OR “4 year old”.ti. OR “5 year old”.ti. OR “6 year old”.ti. OR “two years old”.ti. OR “three years old”.ti. OR “four years old”.ti. OR “five years old”.ti. OR “six years old”.ti. OR “2 years old”.ti. OR “3 years old”.ti. OR “4 years old”.ti. OR “5 years old”.ti. OR “6 years old”.ti.) NOT (exp animals/NOT exp humans/)
**d.) AJOL Search Strategy** ‘schistosomiasis AND children’
**e.) LILACS search Strategy** schistosomiasis AND (children OR Preschool child*)

Published grey literature, WHO literature databases and reports, and documentation obtained from schistosomiasis experts working in relevant organizations such as Kenya Medical Research Institute (KEMRI), Schistosomiasis Control Initiative Foundation (SCIF), Schistosomiasis Consortium for Operational Research and Evaluation (SCORE), the Foundation for Innovative New Diagnostics (FIND), Sight Savers, Hellen Keller and Merck KGaA will also be included in this review. Experts will be actively contacted with an invitation to share relevant documents and references.

All identified references will be screened independently by two reviewers (Phyllis Munyiva Isaiah and Marta Palmeirim) using a two-stage approach.

First, a manual search will be conducted on the initial hit list by reviewing the title and abstracts to identify schistosomiasis studies conducted among PSAC. Second, we will review the full texts of the shortlisted articles to identify studies conducted in hard-to-reach areas and populations.

Additional references will then be retrieved by manually searching the bibliographies of identified articles. All relevant literatures will be downloaded into EndNote X9 to maintain and manage citation and facilitate the overall review process.

*Inclusion/exclusion criteria*

We will include cross-sectional, cohort and case control studies on schistosomiasis in children under 7 years old and living in hard-to-reach areas or belonging to hard-to-reach populations. Similar studies done on women of reproductive age and adults or studies that did not apply cross-sectional or cohort or case control design (e.g. case reports) will be excluded from the review.

### Risk of bias and quality assessment

Reviewers will assess all included studies independently for possible bias by using the Joanna Briggs Institute (JBI) Prevalence Critical Appraisal Tool. ^23^ All selected studies will be assessed using the 10 quality control items suggested by the tool. A score of 1 will be awarded for each item fulfilled while a 0 score will be awarded for each unfilled control item. Score aggregates will then be generated and converted into a low, moderate and high quality classification. ^24^ Poor quality studies will be excluded, clearly documenting the reason for exclusion.

### Data extraction and synthesis

Data will be extracted from included documents and exported to a predefined summary template in Microsoft excel 2016 ( Table 3). Extracted data will include:

i. Bibliographic information (first author, journal/document, year of publication)
ii. Year and country of study
iii. Sample size
iv. Study population age and sex
v. Prevalence and/or incidence of schistosomiasis
vi. Mean eggs per gram of feces (in the case of *S. mansoni*) or per ml of urine (in the case of *S. haematobium*) or infection intensity classification (if available)
vii. Specific schistosome species observed
viii. Diagnostic method
ix. Type of hard-to-reach population/area

**Table 3.** Data extraction tool.

Author name and year	Study country	Study population age	Sample size	Study population sex	Schistosomiasis positive cases	Schistosomiasis prevalence (%)	Mean eggs per gram of stool (epg) or Mean eggs per ml urine	Infection intensity classification	Schistosome species	Diagnostic approach	Type of hard-to- reach population/area	Sampling strategy


### Data analysis

Extracted data will be analyzed using IBM SPSS statistics V.24. Descriptive statistics will be performed to allow for narrative synthesis. Weighted population mean outcomes will then be calculated for prevalence among PSAC. ^25^ To calculate pooled prevalence estimates (PPE), the inverse variance heterogeneity (IVhet) model ^26^ in MetaXL will be used for the selected studies, to ensure that statistical error is not underestimated. ^24^ The level of heterogeneity on selected studies will be evaluated using Cochran’s Q and *I ^2^* statistics. This will be done by stratifying our data according to schistosomiasis prevalence and the region where the studies were conducted to determine heterogeneity between subgroups and within-groups. ^24^ Forest plots will be used to show the estimated prevalence (95% confidence interval).

### Dissemination of results

All findings will be published in a scientific article in a peer reviewed journal. The findings of this review will be reported in accordance with Preferred Reporting Items for Systematic Reviews and Meta-Analyses–Extension for Scoping Reviews (PRISMA-ScR) guidelines.

### Study status

Electronic databases have been searched using the above-mentioned search strategy for articles. The reviewers are currently screening the articles for duplicates.

## Data availability

No data are associated with this article.
