# Supplementary material for: Epidemiology of pediatric schistosomiasis in hard-to-reach areas and populations: A scoping review protocol
Source: F1000Res. 2023 Jan 20;11:1203. Originally published 2022 Oct 21. [Version 2] doi: 10.12688/f1000research.126884.2 (PMC9898687; doi:10.12688/f1000research.126884.2)
Supplement: Peer review history

# Peer review: Reviewer response for version 2

**DOI:** 10.5256/f1000research.143202.r161017

**Published:** 2023-02-03

**Authors:** Dirk Engels

**Affiliations:** 1 Uniting to Combat NTDs Support Centre, Geneva, Switzerland

I have seen the clarifications by the authors and they are sufficient for me to amend my previous status to “Approved”

Is the study design appropriate for the research question?

Yes

Is the rationale for, and objectives of, the study clearly described?

Yes

Are sufficient details of the methods provided to allow replication by others?

Yes

Are the datasets clearly presented in a useable and accessible format?

Yes

Reviewer Expertise:

NA

I confirm that I have read this submission and believe that I have an appropriate level of expertise to confirm that it is of an acceptable scientific standard.

# Peer review: Reviewer response for version 2

**DOI:** 10.5256/f1000research.143202.r161018

**Published:** 2023-01-27

**Authors:** Zhou Xiao-Nong

**Affiliations:** 1 Chinese Center for Disease Control and Prevention, National Institute of Parasitic Diseases, Shanghai, China; 2 School of Global Health, Chinese Center for Tropical Dsieases Research, Shanghai Jiao Tong University School of Medicine, Shanghai, China

All my comments have been addressed well, but two minor issues need to be revised.

1. Under the Introduction, the sentence of "Estimates indicate that schistosomiasis causes an annual loss of 4.5 million disability-adjusted life years (DALYs)" need to check a proper reference on the number of the DALYs, so the number of DALYs may be changed?
2. In the Table 1, "Schistosomiasis infection" changes as "Schistosomes infection" or " *Schistosoma* spp. infection".

Is the study design appropriate for the research question?

Partly

Is the rationale for, and objectives of, the study clearly described?

Yes

Are sufficient details of the methods provided to allow replication by others?

Partly

Are the datasets clearly presented in a useable and accessible format?

Partly

Reviewer Expertise:

Epidemiolgoy of schistosomiasis

I confirm that I have read this submission and believe that I have an appropriate level of expertise to confirm that it is of an acceptable scientific standard.

# Peer review: Reviewer response for version 1

**DOI:** 10.5256/f1000research.139341.r156448

**Published:** 2023-01-10

**Authors:** Zhou Xiao-Nong

**Affiliations:** 1 Chinese Center for Disease Control and Prevention, National Institute of Parasitic Diseases, Shanghai, China; 2 School of Global Health, Chinese Center for Tropical Dsieases Research, Shanghai Jiao Tong University School of Medicine, Shanghai, China

The authors are trying to review epidemiology of pediatric schistosomiasis in hard-to-reach areas and populations, the results will provide evidences to designing the extension of preventive chemotherapy (PC) programs in the global schistosomiasis control program, by using the five-stage scoping review process. It is worthwhile for publication of the protocol due to big gaps are existed in the previous global NTDs program that focus only on the population over 6 years old. But there are several limitations to design the protocol based on the objectives of the study, which can overcome by revision of the study protocol.

1. The scope of the population to be studied: based on the objectives to provide evidence to designing the extension of PC program, it is essential to understand the current situation of all pre-school age children (PSAC) versus PSAC living in the hard-to-reach areas. Therefore, in the protocol, it is suggested to include the data from whole PSAC population, then to compare the epidemic features between PSAC living in the hard-to-reach areas and all of PSAC in endemic areas.
2. The definition of hard-to-reach areas and hard-to-reach population should be different, but the authors only provide the definition of hard-to-reach population, which covers three types of features. For my understanding, the PSAC population living in the hard-to reach areas is the part of hard-to-reach population. In addition, those living in rich areas but always in the migrate status, such as boat family, etc. So it is suggested to give definitions both of hard-to-reach areas as well as hard-to-reach populations.
3. In the objective part, it is suggested to firstly provide a general objective with two to three specific aims, to indicate detail objectives of the study designing. Then followed by the definitions and scope of the review, in order to clearly describe the final outcomes of the study.
4. In the Methods part, it is suggested to provide the detail information of the data analysis, due to there is vague information of the statistical analysis approach provided in the current version. It is also suggested to provide the data collection tables as attached file, which is very important for researchers to collect data and form the datasets as well as to perform the data handling by using unified SOPs in different countries where studying.
5. Under the Methods part, data extraction and synthesis, point vi. “Mean eggs per gram f feces or infection intensity classification (if available) “ is only suitable for *S. mansoni* infection, but not suitable for *S. haematobium*. So please revise this sentence by consideration of both species of schistosomes.

Is the study design appropriate for the research question?

Partly

Is the rationale for, and objectives of, the study clearly described?

Yes

Are sufficient details of the methods provided to allow replication by others?

Partly

Are the datasets clearly presented in a useable and accessible format?

Partly

Reviewer Expertise:

Epidemiolgoy of schistosomiasis

I confirm that I have read this submission and believe that I have an appropriate level of expertise to confirm that it is of an acceptable scientific standard, however I have significant reservations, as outlined above.

# Response

**Published:** 2023-01-13

**Authors:** Phyllis Isaiah

**Affiliations:** Swiss Tropical and Public Health Institute, Switzerland

**Question 1: ** The scope of the population to be studied: based on the objectives to provide evidence to designing the extension of PC program, it is essential to understand the current situation of all pre-school age children (PSAC) versus PSAC living in the hard-to-reach areas. Therefore, in the protocol, it is suggested to include the data from whole PSAC population, then to compare the epidemic features between PSAC living in the hard-to-reach areas and all of PSAC in endemic areas.

***Response***: Thank you for the feedback.

 A recent study by Kalinda *et al.,* 2020 conducted a systematic review of schistosomiasis infection burden among PSAC. We also anticipate that in view of the availability of pediatric praziquantel, intervention need will shift to ensure the very marginalized PSAC are covered, hence the focus on hard-to-reach areas and population. We have however compared the results of the systematic review by Kalinda and colleagues with our results in the actual scoping review manuscript.

**Question 2:** The definition of hard-to-reach areas and hard-to-reach population should be different, but the authors only provide the definition of hard-to-reach population, which covers three types of features. For my understanding, the PSAC population living in the hard-to reach areas is the part of hard-to-reach population. In addition, those living in rich areas but always in the migrate status, such as boat family, etc. So it is suggested to give definitions both of hard-to-reach areas as well as hard-to-reach populations.

***Response:*** Thank you for pointing this out. This has been amended in the protocol.

**Question 3:** In the objective part, it is suggested to firstly provide a general objective with two to three specific aims, to indicate detail objectives of the study designing. Then followed by the definitions and scope of the review, in order to clearly describe the final outcomes of the study.

***Response:*** Thank you for pointing this out. This has been amended in the protocol. We had only one objective.

**Question 4:** In the Methods part, it is suggested to provide the detail information of the data analysis, due to there is vague information of the statistical analysis approach provided in the current version. It is also suggested to provide the data collection tables as attached file, which is very important for researchers to collect data and form the datasets as well as to perform the data handling by using unified SOPs in different countries where studying.

***Response:*** Thank you for pointing this out. This has been amended in the protocol. An annex (a data collection table) has been attached.

**Question 5:** Under the Methods part, data extraction and synthesis, point vi. “Mean eggs per gram f feces or infection intensity classification (if available) “ is only suitable for *S. mansoni* infection, but not suitable for *S. haematobium*. So please revise this sentence by consideration of both species of schistosomes.

***Response:*** Thank you for pointing this out. This has been amended in the protocol.

# Peer review: Reviewer response for version 1

**DOI:** 10.5256/f1000research.139341.r156449

**Published:** 2022-12-15

**Authors:** Dirk Engels

**Affiliations:** 1 Uniting to Combat NTDs Support Centre, Geneva, Switzerland

The study is relevant as it is clearly important, especially in view of the ADOPT programme for pediatric praziquantel, to have best estimates of the global number of pre-school age children to be reached. My main remark would be: with such a substantial scoping review, why limiting the study to hard-to-reach populations only? I suggest to expand to all eligible studies reporting on schistosomiasis in PSAC and treat hard-to-reach populations as a sub-analysis. In this way as-broad-as-possible information is captured in one scoping review, leaving all options open for an access plan

Is the study design appropriate for the research question?

Yes

Is the rationale for, and objectives of, the study clearly described?

Yes

Are sufficient details of the methods provided to allow replication by others?

Yes

Are the datasets clearly presented in a useable and accessible format?

Yes

Reviewer Expertise:

Tropical Diseases

I confirm that I have read this submission and believe that I have an appropriate level of expertise to confirm that it is of an acceptable scientific standard, however I have significant reservations, as outlined above.

# Response

**Published:** 2023-01-13

**Authors:** Phyllis Isaiah

**Affiliations:** Swiss Tropical and Public Health Institute, Switzerland

***Question 1:*** The study is relevant as it is clearly important, especially in view of the ADOPT programme for pediatric praziquantel, to have best estimates of the global number of pre-school age children to be reached. My main remark would be: with such a substantial scoping review, why limiting the study to hard-to-reach populations only? I suggest to expand to all eligible studies reporting on schistosomiasis in PSAC and treat hard-to-reach populations as a sub-analysis. In this way as-broad-as-possible information is captured in one scoping review, leaving all options open for an access plan

***Response***: Thank you for the feedback.

 A recent study by Kalinda *et al.,* 2020 conducted a systematic review of schistosomiasis infection burden among PSAC. We also anticipate that in view of the availability of pediatric praziquantel, intervention need will shift to ensure the very marginalized PSAC are covered, hence the focus on hard-to-reach areas and population. We have however compared the results of the systematic review by Kalinda and colleagues with our results in the actual scoping review manuscript.
